# Food insecurity among older adult Asian Americans: concerning trends

**DOI:** 10.1017/S1368980025100979

**Published:** 2025-08-27

**Authors:** Lilly Nhan, Lisa G. Rosas, Lan Xiao, Wei-ting Chen, May Wang

**Affiliations:** 1 Department of Community Health Sciences, Fielding School of Public Health, University of California, Los Angeles, CA 90095, USA; 2 Department of Epidemiology and Population Health, Stanford School of Medicine, Stanford, CA 94305, USA; 3 Department of Medicine, Division of Primary Care and Population Health, Stanford School of Medicine, Stanford, CA 94305, USA

**Keywords:** Asian Americans, Food insecurity, Elderly, Disaggregated data, SNAP, Ethnic subgroups

## Abstract

**Objective:**

Little is known about food insecurity in Asian Americans (AA). We examined age/ethnic subgroup differences in food insecurity among AA in California.

**Design:**

We examined associations between food insecurity and socio-demographic characteristics among AA (Chinese, Filipino, Korean, and Vietnamese) using the *χ* ^2^ test. Rolling averages were calculated to examine food insecurity trends.

**Setting:**

California.

**Participants:**

We used data from the California Health Interview Survey (2011–2018) for AA categorised by age (18–39, 40–59 and 60+ years).

**Results:**

Food insecurity prevalence varied by subgroup, with the highest observed in older adult (aged 60+ years) Vietnamese (26 %). Between 2011–2014 and 2015–2018, food insecurity prevalence increased 20–45 % across older adults, but showed a decreasing trend among younger adults. Being foreign born and speaking a language other than English at home were associated with increased food insecurity.

**Conclusions:**

Community-engaged research to develop culturally appropriate strategies for mitigating food insecurity among older AA is warranted.

Food insecurity remains a serious public health problem. In 2019, 11 % of U.S. households suffered from food insecurity^(1)^, defined as the limited and uncertain acquisition of nutritionally adequate foods through socially acceptable ways^(2)^. The burden of household food insecurity has been disproportionately borne by racial/ethnic minority groups, with food insecurity prevalence for Black and Hispanic households hovering at 22 % and 17 % in 2020, respectively^(1,3)^.

Little is known about the prevalence of food insecurity among Asian Americans (AA) whose population grew by 35·5 % over the past decade^(4)^. Data on AA are usually aggregated, ignoring the diversity of experiences of AA subgroups and potentially masking ethnic subgroup differences^(5)^. The few published studies of food insecurity among AA report varying prevalence among ethnic subgroups, consistent with the heterogeneity of income among Asian ethnic subgroups, which is greater than that of other racial/ethnic groups^(6)^. Pooling data from the California Health Interview Survey (CHIS) over 10 years, Becerra *et al.* examined ethnic subgroup differences in food insecurity among AA adults and found wide variation in prevalence, ranging from 2·3 % among Japanese to 16·4 % among Vietnamese^(6)^. Not speaking English at home was associated with increased food insecurity risk among Chinese, Koreans and Vietnamese but not Filipinos or Japanese. Adults aged 45+ years had significantly higher prevalence of food insecurity compared with younger adults (18–44 years) among Chinese, South Asian, Korean and Vietnamese subgroups. In another study, Louie *et al.* studied a convenience sample of sixty-eight California-residing Asians and Pacific Islanders and found 60 % were food insecure but only 30 % had ever applied for CalFresh (California’s Supplemental Nutrition Assistance Program) benefits. Shame and pride and lack of knowledge about eligibility requirements were cited as primary reasons^(7)^.

These existing studies examining food insecurity among AA do not report results specific to older adults (ages 60+ years). Older adults are uniquely vulnerable to food insecurity given a high prevalence of chronic disease, physical and cognitive limitations and fixed income^(8)^. Having multiple chronic diseases is associated with higher risk of food insecurity; strained household budgets from increased healthcare expenses may partially explain this observation^(9)^. Ageing also increases the risk for physical and cognitive limitations, which may impact an older adult’s ability to perform daily living activities, such as purchasing and preparing food, increasing food insecurity risk^(10)^. Older adults are more likely to live on fixed incomes, limiting how much they can spend on food when prices increase with inflation.

This study uses CHIS data to compare food insecurity prevalence across age groups among AA ethnic subgroups residing in California^(11)^. The goal is to inform the implementation of food assistance programs and policies especially during the recovery years of the pandemic, while also filling a gap in the literature on food insecurity in older adult AA, a group reported by the media to have been seeking food assistance and the subject of anti-Asian attacks during the pandemic^(12,13)^.

## Methods

### Data source and study population

CHIS is the nation’s largest state health survey^(11)^. Starting in 2001 as a biennial phone survey conducted in multiple languages, CHIS used a dual-frame random-digit-dial sampling technique prior to 2019^(14)^. Currently, it uses an address-based sampling frame and is a phone and web-based survey of over 20 000 households per year that is conducted on a continuous basis. The survey is conducted in six languages (English, Spanish, Chinese (Mandarin and Cantonese dialects), Vietnamese, Korean and Tagalog). We used 2-year public use data files from 2011 to 2018 for adults and AA subgroups with sample sizes that allowed for stable statistical estimates of food insecurity rates, specifically, Chinese, Korean, Filipino and Vietnamese.

### Measurements

#### Food insecurity

Food insecurity was assessed using the six-item USDA food security survey module which used Likert scale or yes/no responses to assess agreement with statements such as ‘The food that (I/we) bought just didn’t last, and (I/we) didn’t have money to get more’ ‘Often true’ and ‘sometimes true’ or ‘yes’ were deemed affirmative responses^(15)^. Food security was operationalised as having no more than one affirmative response while food insecurity was operationalised as having two or more affirmative responses. The food security module was administered only to households with income ≤ 200 % of the federal poverty level (FPL) or unknown income. Food insecurity prevalence was calculated as the percent of all respondents (including those with income > 200 % FPL) who were assessed as food insecure.

#### Socio-demographic characteristics

Socio-demographic characteristics included the following: whether the respondent was born in the USA, citizenship status; language spoken at home, income expressed as percent of the FPL, household size, housing, educational attainment, employment status, health insurance, participation in SNAP/CalFresh and receiving supplemental security income or social security disability insurance (SSI/SSDI).

### Statistical analysis

We examined three age groups: 18–39, 40–59 and 60+ years. Unweighted counts and weighted percentages were generated from PROC SURVEYFREQ. Weighted means were generated from PROC SURVEYMEANS. Associations between food insecurity and socio-demographic characteristics were assessed using the *χ* ^2^ test for each ethnic and age group. To examine trends in food insecurity prevalence over time, rolling averages were calculated for each ethnic and age group across four-year periods to allow for adequate sample sizes in each ethnic/age group (2011–2014, 2013–2016 and 2015–2018). To assess the precision of each point estimate, we calculated the CV, which is the se divided by the point estimate^(16)^. We identified any point estimates with a CV > 0·3 in the results as potentially statistically unstable, as recommended by CHIS^(17)^. All analyses were conducted using SAS, version 9.4 (SAS Institute Inc.) and took account of the complex sampling design and sample weights of CHIS. Statistical significance was set at *P* < 0·05 (two-sided). This study was determined to be exempt from review by the University of California, Los Angeles Institutional Review Board.

## Results

### Socio-demographic characteristics

Ethnic subgroup differences in socio-demographic characteristics are presented in Table 1. Education levels were similar among Chinese, Korean and Filipino with over half reporting having a college degree or higher but lower for Vietnamese with 52 % reporting having only a high school diploma or lower. They were also generally lowest among older adults. The majority were born outside of the USA; older respondents were more likely to be born outside the USA than younger respondents. A high proportion of Chinese (43 %), Korean (45 %) and Vietnamese (58 %) respondents reported they spoke only their native language at home, with the oldest age group reporting the highest proportions. About a quarter to a third of Filipino, Chinese and Korean and over half of Vietnamese respondents were classified as having household income ≤ 200 % FPL. Poverty levels were highest among older adults in all ethnic subgroups.

**Table 1.** Study population characteristics by age and Asian subgroup, California Health Interview Survey 2011–2018^†^

	18–39 years								40–59 years								60+ years								18+ years							
	Chinese		Korean		Filipino		Vietnamese		Chinese		Korean		Filipino		Vietnamese		Chinese		Korean		Filipino		Vietnamese		Chinese		Korean		Filipino		Vietnamese	
	*n*	%	*n*	%	*n*	%	*n*	%	*n*	%	*n*	%	*n*	%	*n*	%	*n*	%	*n*	%	*n*	%	*n*	%	*n*	%	*n*	%	*n*	%	*n*	%
Food insecurity^‡^ (%)																																
Food insecure	69	4·7	25	5^*^	88	8·1	46	9·2	109	5·7^*^	33	5·5^*^	93	10·8	148	15·4^*^	250	17	163	10·9^*^	112	14·8	283	25·7	428	7·5	221	6·8^*^	293	10·4	477	16·1
Food secure	298	22·3	94	25·2	141	14·9	159	29·5	253	16·1	98	15·9	96	10·2	292	36·1	636	30·3^*^	549	43·3	200	28·2	676	50·3	1187	21·8	741	27·4	437	16·2	1127	37·8
Not applicable	932	73·1	264	69·7	582	77	249	61·3	1314	78·3	428	78·6	606	79	322	48·5	1155	52·7	385	45·8^*^	491	57	224	24	3401	70·7	1077	65·8	1679	73·5	795	46·2
Education level (%)																																
High school or less	315	19·9	108	23·9	247	24·1	177	31·8	305	24·9^*^	116	18·8	102	9·2	405	57·2	660	48	501	43·7	156	23	800	70	1280	27·4	725	27·9	505	19·2	1382	51·6
Some college	206	15·7	65	16	230	30	94	19·5^*^	183	9	78	15·9	200	26·6	136	14·2^*^	264	10·1	120	9·9^*^	166	18·5	163	12·5	653	12·3	263	14·2	596	26·6	393	15·7^*^
Bachelors or more	778	64·4	210	60·1	334	45·8	183	48·7	1188	66	365	65·3	493	64·2	221	28·6	1117	41·9	476	46·5	481	58·5	220	17·5	3083	60·4	1051	57·9	1308	54·3	624	32·8
Born in US (%)																																
Yes	643	47	187	44·6	519	62·2	179	43·3	357	17·5	55	6·9^*^	228	24	7	1^*^	384	12·4	22	3·6^*^	119	9·6	2	0·1^*^	1384	29·8	264	21·2^*^	866	39·2	188	16·2
No	656	53	196	55·4	292	37·8	275	56·7	1319	82·5	504	93·1	567	76	755	99	1657	87·6	1075	96·4	684	90·4	1181	99·9	3632	70·2	1775	78·8	1543	60·8	2211	83·8
Citizenship status (%)																																
Yes	975	74·1	277	67·7	706	84·9	376	87·8	1402	77·7	362	54·3	653	81·9	633	82·5	1898	89·3	986	88·3	739	90·3	1094	88·6	4275	78·5	1625	69·3	2098	85·1	2103	86·2
No	324	25·9	106	32·3	105	15·1	78	12·2	274	22·3^*^	197	45·7	142	18·1	129	17·5	143	10·7	111	11·7^*^	64	9·7	89	11·4	741	21·5	414	30·7	311	14·9	296	13·8
Language spoken at home (%)																																
English only	326	21·1	97	20·9^*^	459	52·8	62	16·6^*^	409	19·8	103	20^*^	345	42	43	5·9^*^	377	13·2^*^	39	6·5^*^	174	16·6	8	1·5^*^	1112	19·1	239	16·6^*^	978	41·8	113	8·5
English and Other language	545	45·8	193	58·4	307	40·7	228	50·7	663	38·2	180	27·2^*^	359	46·8	227	30·8	531	21·1^*^	233	23^*^	466	60·7	168	14·7	1739	38·1	606	38·6	1132	46·8	623	33·5
Other language only	428	33·1	93	20·7^*^	45	6·6^*^	164	32·7	604	42	276	52·8	91	11·2	492	63·3	1133	65·6	825	70·4	163	22·7	1007	83·8	2165	42·8	1194	44·8^*^	299	11·4	1663	58
Percent of federal poverty level (%)																																
0–99 %	165	12·5	68	17·4	89	7·2	117	20·6	158	7·9	45	6·1^*^	81	8·9	260	26·2	478	22·9^*^	400	24·3^*^	140	20	552	43·6	801	13	513	15·8	310	10·4	929	29·1
100–199 %	203	14·4	52	12·9^*^	140	15·8	88	18·1^*^	205	13·9	86	15·3	108	12·1	181	25·4	411	24·5	314	30·4	174	23·3	407	32·4	819	16·3	452	18·6	422	16·2	676	24·7
200–299 %	175	14·1	59	19·6^*^	138	16·7	58	11·5	164	11·7	82	15·9^*^	98	12·6	70	11·9	241	13·4^*^	122	14·6^*^	108	13·9	89	6·8^*^	580	13·1	263	17	344	14·8	217	10·3
300 % and above	756	58·9	204	50·1	444	60·3	191	49·8	1149	66·6	346	62·7	508	66·4	251	36·5	911	39·2	261	30·8^*^	381	42·8	135	17·2^*^	2816	57·5	811	48·6	1333	58·6	577	35·8
	Mean	se	Mean	se	Mean	se	Mean	se	Mean	se	Mean	se	Mean	se	Mean	se	Mean	se	Mean	se	Mean	se	Mean	se	Mean	se	Mean	se	Mean	se	Mean	se
Household size	3·4	0·2	3·1	0·1	4·0	0·1	3·8	0·1	3·5	0·2	3·4	0·2	3·8	0·1	4·2	0·3	2·6	0·2	2·3	0·4	3·0	0·2	3·2	0·2	3·3	0·05	3·0	0·1	3·7	0·1	3·7	0·1
	*n*	%	*n*	%	*n*	%	*n*	%	*n*	%	*n*	%	*n*	%	*n*	%	*n*	%	*n*	%	*n*	%	*n*	%	*n*	%	*n*	%	*n*	%	*n*	%
Own or rent home (%)																																
Own	657	55	142	42·2	337	49	192	50·3	1277	80·1	323	55·1	461	62·9	358	57·5	1269	60·6	420	49·3^*^	482	60·7	261	33·4	3203	64·8	885	48·2	1280	55·8	811	47·9
Rent	544	39·9	221	56	415	44·8	235	47	339	18·8^*^	227	44·3	283	32·7	364	40·9	609	32·7	585	44^*^	258	31·1	798	58·9	1492	31·2	1033	48·9	956	38·1	1397	48·3
Other arrangement	74	5·1	15	1·9^*^	51	6·2	17	2·7^*^	31	1·1^*^	6	0·6^*^	40	4·4	20	1·6^*^	121	6·8^*^	73	6·7^*^	51	8·1	108	7·8^*^	226	4	94	2·8^*^	142	6·1	145	3·8
Employment status (%)																																
Employed	897	71·2	252	69	576	71·3	289	73	1320	82·8	407	77·5	630	84·4	476	74·2	471	28·1	168	25·1	214	29·5	155	17·6	2688	66·4	827	59·2	1420	66·8	920	57·5
Unemployed and looking for work	113	7·9^*^	31	7·8^*^	101	13	52	11·1^*^	73	3·9	20	3·8^*^	49	6·4	71	6·5	35	2·2^*^	15	3^*^	20	3·6^*^	20	2·1^*^	221	5·4	66	5·2^*^	170	9	143	6·9^*^
Unemployed and not looking for work	289	20·9	100	23·2	134	15·6	113	16	283	13·3	132	18·7	116	9·1	215	19·3	1535	69·7	914	71·9	569	66·9	1008	80·3	2107	28·3	1146	35·5	819	24·3	1336	35·6
Currently has health insurance (%)																																
Yes	1140	84·1	308	83·3	709	89·4	388	87·5	1557	93	423	74·9	729	91·5	679	85·6	1971	95·1	1055	93·6	777	95·4	1140	97·2	4668	89·4	1786	83·6	2215	91·3	2207	89·6
No	159	15·9	75	16·7^*^	102	10·6	66	12·5	119	7^*^	136	25·1	66	8·5	83	14·4	70	4·9	42	6·4^*^	26	4·6^*^	43	2·8^*^	348	10·6	253	16·4	194	8·7	192	10·4
Enrolled in SNAP^§^ (%)																																
Yes	48	7·2^*^	6	2·8^*^	33	6·8^*^	61	17·1^*^	49	7·3^*^	17	6·6^*^	37	8·2^*^	150	18·9	80	6·8^*^	49	7^*^	38	10·4	108	13·8^*^	177	7·1	72	5·3^*^	108	8·2^*^	319	16·5
No	540	92·8	180	97·2	346	93·2	221	82·9	555	92·7	223	93·4	267	91·8	375	81·1	1113	93·2	822	93	411	89·6	950	86·2	2208	92·9	1225	94·7	1024	91·8	1546	83·5
Receiving supplemental security income or supplemental security disability insurance^||^ (%)																																
Yes	21	1·1^*^	8	2^*^	17	1·6^*^	8	1·6^*^	43	1·6^*^	19	1^*^	41	2·3	52	4·9^*^	450	21·4^*^	467	31·7^*^	113	14·1	626	43·1	514	5·4^*^	494	10·1^*^	171	4·4	686	14·6
No	1278	98·9	375	98	794	98·4	446	98·4	1633	98·4	540	99	754	97·7	710	95·1	1591	78·6	630	68·3	690	85·9	557	56·9	4502	94·6	1545	89·9	2238	95·6	1713	85·4

* Indicates statistically unstable estimates with CV > 0·3.

† Analyses conducted using survey weights provided by California Health Interview (CHIS). Frequencies are unweighted and proportions are weighted. Means and se are weighted.

‡ Food insecurity was only assessed among those with incomes </= 200 % FPL; not applicable indicates those above 200 % FPL.

§ Supplemental Nutrition Assistance Program (SNAP) enrollment was only assessed among those with incomes </= 300 % FPL.

|| Supplemental Security Income (SSI)/Supplemental Security Disability Insurance (SSDI) receipt was only assessed among those with incomes </= 300 % FPL.

Among all adults, food insecurity prevalence was highest among Vietnamese (16 %) followed by Filipino (10 %) with lower levels among Chinese (7 %) and Korean (7 %) (Table 1). For every ethnic subgroup, food insecurity prevalence was highest among older adults with especially high levels among Chinese (17 %) and Vietnamese (26 %). Among all adults, food insecurity prevalence was significantly higher among those with lower education, born outside of the USA, without U.S. citizenship, who spoke a language other than English at home, with the lowest incomes (0–99 % FPL), who were unemployed, and without health insurance (Table 2). Dose–response effects were observed for education and language spoken at home for all age/ethnic subgroups. U.S. citizenship, poverty level and health insurance were not associated with food insecurity among older adults.

**Table 2.** Association between prevalence of food insecurity and study population characteristics among Asian Americans by age, California Health Interview Survey 2011–2018^†^

	18–39 years		40–59 years		60+ years		18+ years	
	Food insecurity prevalence	se	Food insecurity prevalence	se	Food insecurity prevalence	se	Food insecurity prevalence	se
Education level	**<0·0001**		**<0·0001**		**<0·0001**		**<0·0001**	
High school or less	9·4	3·3^*^	20·0	2·3	25·3	2·6	18·0	1·4
Some College	9·2	1·8	10·0	3·2^*^	16·7	4·0	10·7	1·4
Bachelors or more	4·3	0·9	4·1	1·0	8·8	2·1	5·0	0·7
Born in US	**0·01**		**0·001**		**0·0009**		**0·001**	
Yes	5·4	0·9	3·0	1·9^*^	2·5	2·3^*^	4·8	0·7
No	7·8	1·7	10·0	1·5	18·3	1·8	11·8	1·0
Citizenship status	0·0004		<0·0001		0·2		0·0006	
Yes	5·3	0·8	6·7	2·0	16·4	2·6	8·6	1·3
No	11·2	3·1	15·8	2·7	22·2	10·4^*^	14·4	3·0
Language spoken at home	**<0·0001**		**<0·0001**		**<0·0001**		**<0·0001**	
English only	4·6	1·7^*^	3·9	2·1^*^	4·6	2·2^*^	4·4	0·7
English and Other language	7·2	1·2	7·6	1·3	13·4	2·7	8·5	0·8
Other language	8·0	1·9	13·6	2·4	21·5	2·5	15·0	1·3
Percent of federal poverty level	0·34		0·38		0·25		**0·02**	
0–99 %	26·4	4·0	40·4	10·5	36·0	5·7	33·6	2·3
100–199 %	21·9	3·2	29·2	4·3	28·9	3·0	26·3	1·9
Employment status	**<0·0001**		**<0·0001**		**0·02**		**<0·0001**	
Employed	5·3	0·8	7·2	1·3	9·6	2·2	6·5	0·6
Unemployed and looking for work	10·8	3·0	22·6	7·8^*^	27·8	14·2^*^	15·3	2·8
Unemployed and not looking for work	9·2	3·3^*^	13·7	2·9	19·4	2·4	15·6	1·5
Currently has health insurance	**<0·0001**		**<0·0001**		0·8		**<0·0001**	
Yes	5·5	1·1	7·4	2·0	16·6	1·4	8·8	0·7
No	13·4	3·1	21·4	4·6	25·6	21·7^*^	17·3	2·5
Enrolled in SNAP^§^	**0·01**		**0·01**		**0·02**		**<0·0001**	
Yes	23·5	8·8^*^	38·8	172^*^	51·9	7·3	37·7	4·4
No	13·8	1·7	19·5	1·9	22·2	1·9	18·0	1·1

CHIS, California Health Interview Survey; FPL, federal poverty level.

Bolded values indicate *P* < 0·05.

‡Food insecurity was only assessed among those with incomes </= 200 % FPL; not applicable indicates those above 200 % FPL.

* Indicates statistically unstable estimates with CV > 0·3.

† Analyses conducted using *χ* ^2^ test with survey weights provided by CHIS.

§ SNAP enrollment was only assessed among those with incomes </= 300 % FPL.

Among those with income ≤ 200 % FPL, Vietnamese respondents aged 40–59 years reported the highest enrollment in SNAP at 19 %, but the majority of respondents across all subgroups were not enrolled in SNAP (Table 1). SNAP enrollment was associated with higher levels of food insecurity (Table 2).

**Figure 1.**
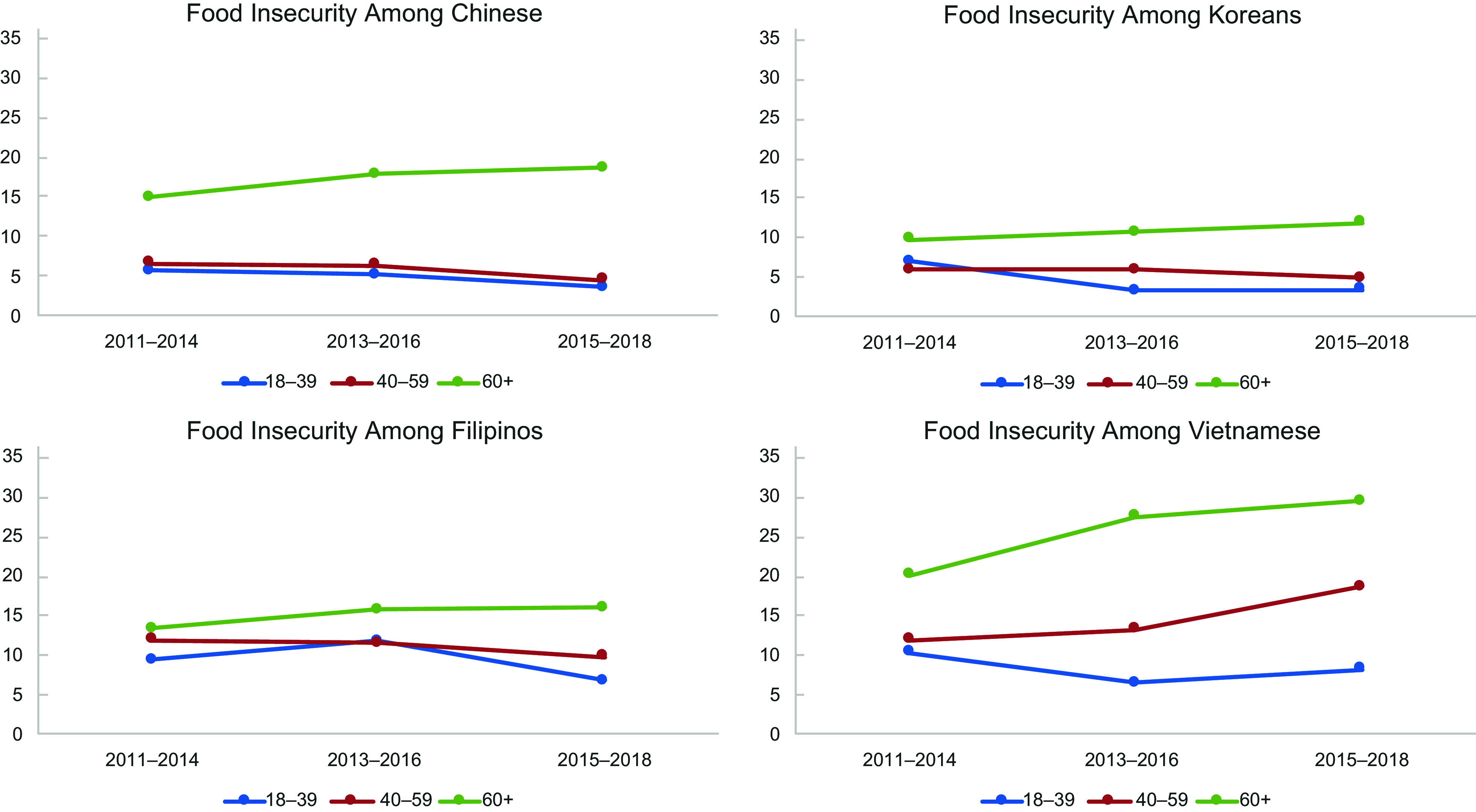
Prevalence of Food Insecurity from 2011 to 2018 by Asian Subgroup and Age, California Health Interview Survey 2011–2018^a^. ^a^ Food insecurity over time calculated using 4-year rolling averages from 2011 to 2018.

Age group differences in food insecurity prevalence varied over the three time periods examined, among ethnic subgroups (Figure 1). The largest gap in food insecurity rates between age groups (higher among older adults) was observed among Chinese followed by Koreans and Filipinos. This gap appears to have increased over time among all three groups due to increasing food insecurity prevalence among the older adults well as decreasing food insecurity rates among most of the younger age groups. Between 2011–2014 and 2015–2018, food insecurity rates among older adults increased by 45 % for Vietnamese, 25 % for Chinese and about 20 % for Filipinos and Koreans. In contrast, they decreased among the two younger age groups for all ethnic subgroups except Vietnamese.

## Discussion

Our study examined differences in food insecurity by age group among AA ethnic subgroups. Determining age group differences in food insecurity has implications for the allocation of limited resources. We found older adults had the highest prevalence of food insecurity and that in some ethnic subgroups, older and younger adults exhibited contrasting trends in food insecurity. The difference in food insecurity prevalence between older adults and young adults was most stark for Chinese. Older adult Chinese had a food insecurity prevalence that was quadruple that for young adult Chinese. Between 2011–2014 and 2015–2018, food insecurity prevalence increased among older adults for every ethnic subgroup, with Vietnamese experiencing a 45 % increase and Chinese, a 25 % increase. In contrast, except for 40- to 59-year-old Vietnamese, food insecurity prevalence decreased over time in each younger age group.

For all adults, the socio-demographic factors associated with increased food insecurity risk in most if not all age/ethnic subgroups were lower education, being foreign born, not being a US citizen, not speaking English at home, being unemployed, not having health insurance and being enrolled in CalFresh (SNAP). This is consistent with the findings from Beccera et al except that they did not include U.S. citizenship, health insurance status and SNAP enrollment in their analysis^(6)^. We found that not having U.S. citizenship and not having health insurance were associated with increased food insecurity among the younger age groups but not older adult AA. Poverty level was not associated with food insecurity, likely due to the fact that the food security questions were only asked of those with incomes <200 % of poverty.

To our knowledge, this is the first study to compare food insecurity rates between older and younger adults for AA ethnic subgroups and report climbing rates of food insecurity among older adult AA in the last decade. However, this study has limitations. First, we had to pool several years of data (to obtain adequate samples), which may mask granular trends in food insecurity. Additionally, due to sample size limitations, we were unable to examine food insecurity among Pacific Islanders who often aggregated with AA; studies suggest food insecurity may be more prevalent in Pacific Islanders than AA^(18)^. Second, CHIS data are only representative for California, so our findings may not be generalisable to other states. Lastly, the data are cross-sectional, limiting our ability to determine causal relationships.

The fastest growing racial group in the country, AA have been overlooked in studies of food insecurity. Our finding of AA older adults having higher rates of food insecurity are corroborated by media reports of AA seniors seeking assistance at food distribution events during the pandemic^(13)^ and reports of rising poverty rates among older adult AA^(19)^. We also find that those who are not U.S. citizens and those who speak a language other an English at home are more likely to be food insecure and that SNAP, the largest food assistance program in the country, does not reach all who may benefit from the program. Further studies of SNAP/CalFresh enrollment among older adult AA during the COVID-19 pandemic to provide insights into the impact of pandemic-related food policy provisions, which allowed for waivers and flexibilities in the operations of food assistance programs, may be helpful^(20)^. More complete collection and reporting of disaggregated health-related data for AA and Pacific Islanders is needed. Older adult AA and Pacific Islanders are often left out of the conversation about the public health needs and well-being of seniors and of AA and Pacific Islanders. Community-engaged research to develop culturally appropriate strategies for increasing the reach and services of SNAP and senior nutrition programs and access to culturally appropriate foods for AA and Pacific Islander older adults is recommended^(21)^.
